# The stress hyperglycaemia ratio is associated with left ventricular remodelling after first acute ST-segment elevation myocardial infarction

**DOI:** 10.1186/s12872-021-01889-8

**Published:** 2021-02-04

**Authors:** Shuai Meng, Yong Zhu, Kesen Liu, Ruofei Jia, Jing Nan, Maolin Chen, Xuan Lei, Kaiyuan Zou, Zening Jin

**Affiliations:** 1 Department of Cardioloy, Beijing Tiantan Hospital, Capital Medical University, Beijing, 100070 China; 2 Department of Cardiology, Beijing Anzhen Hospital, Capital Medical University, Beijing, 100029 China

**Keywords:** ST-segment elevation myocardial infarction, Left ventricular negative remodelling, Stress hyperglycaemia ratiol, Left ventricular ejection fraction, Left ventricular end-diastolic diameter, Left ventricular end-systolic diameter

## Abstract

**Background:**

Left ventricular negative remodelling after ST-segment elevation myocardial infarction (STEMI) is considered as the major cause for the poor prognosis. But the predisposing factors and potential mechanisms of left ventricular negative remodelling after STEMI remain not fully understood. The present research mainly assessed the association between the stress hyperglycaemia ratio (SHR) and left ventricular negative remodelling.

**Methods:**

We recruited 127 first-time, anterior, and acute STEMI patients in the present study. All enrolled patients were divided into 2 subgroups equally according to the median value of SHR level (1.191). Echocardiography was conducted within 24 h after admission and 6 months post-STEMI to measure left ventricular ejection fraction (LVEF), left ventricular end-diastolic diameter (LVEDD), and left ventricular end-systolic diameter (LVESD). Changes in echocardiography parameters (δLVEF, δLVEDD, δLVESD) were calculated as LVEF, LVEDD, and LVESD at 6 months after infarction minus baseline LVEF, LVEDD and LVESD, respectively.

**Results:**

In the present study, the mean SHR was 1.22 ± 0.25 and there was significant difference in SHR between the 2 subgroups (1.05 (0.95, 1.11) vs 1.39 (1.28, 1.50), *p* < 0.0001). The global LVEF at 6 months post-STEMI was significantly higher in the low SHR group than the high SHR group (59.37 ± 7.33 vs 54.03 ± 9.64, *p*  = 0.001). Additionally, the global LVEDD (49.84 ± 5.10 vs 51.81 ± 5.60, *p*  = 0.040) and LVESD (33.27 ± 5.03 vs 35.38 ± 6.05, *p*  = 0.035) at 6 months after STEMI were lower in the low SHR group. Most importantly, after adjusting through multivariable linear regression analysis, SHR remained associated with δLVEF (beta = −9.825, 95% CI −15.168 to −4.481, *p*  < 0.0001), δLVEDD (beta = 4.879, 95% CI 1.725 to 8.069, *p*  = 0.003), and δLVESD (beta = 5.079, 95% CI 1.421 to 8.738, *p*  = 0.007).

**Conclusions:**

In the present research, we demonstrated for the first time that SHR is significantly correlated with left ventricular negative remodelling after STEMI.

## Introduction

Despite rapid development in reperfusion therapy, primary percutaneous coronary intervention (PCI), modern antithrombotic therapy, and secondary prevention in recent years, the incidence of post-infarction cardiac remodelling and subsequent heart failure remains relatively high [1, 2]. Myocardial infarction, especially ST-segment elevation myocardial infarction (STEMI), could trigger inflammatory and fibrotic responses in both infarcted and non-infarcted areas, contributing to the occurrence of cardiac remodelling and dysfunctional ventricles [3, 4]. Recently, mounting evidence has revealed that stress hyperglycaemia is also strongly associated with prognosis and post-infarction cardiac remodelling even after successful reperfusion therapy [5–7].

Stress hyperglycaemia is defined as transient hyperglycaemia in response to an acute illness or stress reaction [8, 9]. Admission blood glucose (ABG) was used to identify stress hyperglycaemia in most previous studies [10]. However, ABG levels could reflect both acute physiological stress and chronic glycaemic levels, which limits the application of ABG and prompts the search for a better marker reflecting the degree of stress hyperglycaemia [10]. The stress hyperglycaemia ratio (SHR), a novel index of stress hyperglycaemia, was proposed by Roberts et al.. In the study from Roberts et al., SHR was defined as ABG divided by estimated average glucose (eAG) derived from glycated haemoglobin (HbA1c) [11].

Recently, mounting evidence revealed that SHR correlates positively with adverse events following acute myocardial infarction (AMI), stroke, and other acute illnesses [10, 12, 13]. However, the role of SHR in left ventricular remodelling after AMI remains poorly understood. Therefore, it is necessary to investigated the associations between SHR and left ventricular ejection fraction (LVEF) as well as LV geometric parameters.

## Methods

### Study population

From January 2018 to December 2018, we enrolled 127 patients with first-time, acute, anterior STEMI from Beijing Anzhen Hospital and Being Tiantan Hosipital, Capital Medical University. All the patients recruited had to meet the following inclusion criteria: age range from 18 to 80 years, diagnosis of STEMI according to the fourth universal definition of myocardial infarction, infarct-related artery (left anterior descending artery) confirmed through electrocardiography and emergent coronary angiography, only emergent recanalization of left anterior descending artery by experienced interventional cardiologist within 12 h and management of other severe diseased vessels by elective PCI within 1 month if needed. Additionally, the exclusion criteria were as follows: age more than 80 years, prior myocardial infarction, prior history of coronary artery revascularization, rescue angioplasty, chronic failure, cardiomyopathy, hepatic and renal dysfunction, malignancy and the requirement of steroid or immunosuppressive therapy. Most importantly, due to the blood diseases could potentially have effects on measurement of HbA1c, the patients who have been diagnosed with hemolytic anemia, aplastic anemia (AA), myelodysplastic syndromes (MDS), and others were not included in this study.

Our present study got approvement from the Ethics Committee of Beijing Anzhen Hospital and Beijing Tiantan Hospital, Capital Medical University and was performed in line with the Declaration of Helsinki. Written informed consent from all the participants in the present research were obtained by us.

### Procedure and periprocedural management

Before primary PCI, all the participants in the present research were prescribed a loading dose of aspirin 300 mg, clopidogrel 600 mg or ticagrelor 180 mg. In addition to antiplatelet therapy, anticoagulation therapy was recommended to all the participants as well. Furthermore, the primary PCI strategy, the choice of second-generation drug-eluting stents, and the application of thrombus aspiration and glycoprotein IIb/IIIa inhibitors all adhered to the current guidelines and the discretion of the surgeons. After primary PCI, dual antiplatelet therapy (aspirin plus clopidogrel or ticagrelor), statins, beta-blockers, and angiotensin-converting enzyme inhibitors (ACEIs)/angiotensin receptor blockers (ARBs) were all recommended to the participants in addition to lifestyle intervention and risk factor control.

### Baseline clinical data collection

When the participants reached the hospital, we measured ABG immediately with a standardized biochemical assay. We also tested HbA1c in the participants using a liquid chromatography analyser. As reported by Nathan et al., we calculated eAG according to the following equation: eAG (mmol/L) = (1.59 × HbA1c-2.59) [14]. Then, we defined SHR as ABG/eAG in line with previous studies [10–12]. In addition to that, we also monitored cardiac troponin I (cTnI) and creatine kinase muscle/brain subtype (CK-MB) dynamically to identify peak cTnI and peak CK-MB. Routine blood, urine, and biochemical tests were performed in the central laboratory of Beijing Anzhen Hospital and Beijing Tiantan Hospital.

Each patient’s baseline clinical data, such as age, sex, body mass index (BMI, kg/m^2^), Killip class on admission, and personal medical histories, were recorded. Coronary angiogram data including numbers of significantly stenosed vessels, thrombolysis in myocardial infarction (TIMI) flow grade, number of stents, and diameter and length of stents were also collected by us. Furthermore, we also recorded the total ischaemic time and medication use at discharge.

### Echocardiographic assessment

Transthoracic echocardiography was performed within 24 h after admission and at the 6-month follow-up using a GE Vivid E7 ultrasonography machine (GE healthcare, Piscataway, NJ, USA) by 2 independent echocardiographers. The echocardiographers obtained a standard echocardiographic view with the supervision of an experienced cardiologist. Then, the echocardiographers measured LVEF, left ventricular end-systolic diameter (LVESD) and left ventricular end-diastolic diameter (LVEDD) with Simpson’s modified biplane method. Most importantly, we performed all the measurements in accordance with the recommendations of the American Society of Echocardiography and the European Association of Cardiovascular Imaging. Changes in echocardiography parameters (δLVEF, δLVEDD, δLVESD) were calculated as LVEF, LVEDD, and LVESD at 6 months after infarction minus baseline LVEF, LVEDD and LVESD, respectively.

### Statistical analysis

According to the different distributions of variables, continuous variables are summarized as the mean ± standard deviation or the median (lower quartile, upper quartile) and were compared using Student’s t test and Mann–Whitney U test, respectively. Categorical variables are presented as percentages and were compared by Chi-square test or Fisher’s exact test. To further examine the association between SHR and left ventricular remodelling parameters, multivariate linear regression analysis was employed. Age, sex, type 2 diabetes mellitus (T2DM), total ischaemic time, number of diseased vessels, and variables with *p* < 0.05 in univariable linear analysis were all included in the multivariate linear regression model. In the present study, we performed the statistical analyses using SPSS 20.0 (SPSS Inc., Chicago, IL, USA) and *p* < 0.05 was regarded as statistically significant.

## Results

### Baseline and procedural characteristics of patients enrolled

In the present study, the study population was divided into 2 subgroups equally with median value of SHR (1.191). As shown in Tables 1 and 2, the mean SHR in the present study was 1.22 ± 0.25. The average age of the study population was 55.94 ± 11.14 years old, and 86.6% was male. The prevalence of hypertension, T2DM, and current smoking was 52.0%, 40.2%, and 68.5%, respectively. Additionally, 90.6% of STEMI patients presented with Killip class I upon admission, and more than half of STEMI patients had multivessel disease. Furthermore, the total ischaemic time was 422.53 ± 143.43 min, and 94.5% of STEMI patients had TIMI flow ≥ 2 after the intervention, which means almost all the patients recruited in the present study received a timely and successful primary PCI.

**Table 1:** Baseline characteristics of patients enrolled

	Total	Low SHR group (SHR < 1.191)	High SHR group (SHR ≥ 1.191)	*P* value
Number	127	63	64	
Age	55.94 ± 11.14	54.33 ± 11.74	57.52 ± 10.36	0.108
Male sex (%)	110 (86.6%)	58 (92.1%)	52 (81.2%)	0.116
BMI (kg/m^2^)	25.12 ± 2.61	25.48 ± 2.40	24.78 ± 2.78	0.130
Hypertension (%)	66 (52.0%)	32 (50.8%)	34 (53.1%)	0.860
T2DM (%)	51 (40.2%)	20 (31.7%)	31 (48.4%)	0.071
Current smoker (%)	87 (68.5%)	47 (74.6%)	40 (62.5%)	0.182
*Killip classification*				0.405
Class I	115 (90.6%)	55 (87.3%)	60 (93.8%)	
Class II	10 (7.9%)	7 (11.1%)	3 (4.7%)	
Class III	2 (1.6%)	1 (1.6%)	1 (1.6%)	
Class IV	0	–	–	
Baseline LVEF	49.98 ± 8.59	50.84 ± 9.41	49.13 ± 7.67	0.263
*Laboratory examination*				
White blood cells (10^9^/L)	10.45 ± 2.47	10.35 ± 2.60	10.56 ± 2.35	0.639
Hemoglobin (g/L)	153.06 ± 12.96	152.89 ± 13.42	153.22 ± 12.59	0.887
Platelet (10^9^/L)	229.99 ± 63.95	232.79 ± 65.29	227.23 ± 62.99	0.626
eGFR (mmol/L)	99.86 ± 13.44	101.36 ± 14.27	98.38 ± 12.52	0.213
Uric acid (mg/dl)	357.22 ± 92.63	365.91 ± 106.52	348.66 ± 76.46	0.297
ABG (mmol/L)	8.38 (6.95,10.87)	6.99 (6.23,8.48)	10.10 (8.37,11.99)	0.001
HBA_1_C (%)	6.00 (5.60,6.90)	6.00 (5.60,6.90)	6.00 (5.53,6.80)	0.894
SHR	1.22 ± 0.25	1.05 (0.95,1.11)	1.39 (1.28,1.50)	< 0.0001
Triglycerides (mmol/L)	1.43 (1.00,1.97)	1.81 ± 1.13	1.36 (0.90,1.86)	0.116
LDL-C (mmol/L)	3.28 ± 1.06	3.23 ± 1.13	3.33 ± 0.98	0.586
HDL-C (mmol/L)	1.13 ± 0.28	1.06 ± 0.23	1.19 ± 0.31	0.010
Peak CK-MB	122.40 (45.10,258.00)	89.38 (26.60,202.60)	154.60 (54.18,299.00)	0.047
Peak cTnI (ng/ml)	30.62 (12.40,78.00)	20.62 (8.84,40.64)	56.07 (25.43,81.00)	< 0.001
*Medications * (*Discharge)*				
Aspirin (%)	127 (100.0%)	63 (100.0%)	64 (100.0%)	1.000
Clopidogrel/ticagrelor (%)	127 (100.0%)	63 (100.0%)	64 (100.0%)	1.000
Statin (%)	123 (96.9%)	63 (100.0%)	60 (93.8%)	0.119
Beta-blockers (%)	109 (85.8%)	56 (88.9%)	53 (82.8%)	0.446
ACEI/ARB (%)	89 (70.1%)	43 (68.3%)	46 (71.9%)	0.701
Insulin (%)	7 (5.5%)	2 (3.2%)	5 (7.8%)	0.440
Hypoglycemic agents (%)	30 (23.6%)	13 (20.6%)	17 (26.6%)	0.532

BMI, body mass index; T2DM, type 2 diabetes mellitus; LVEF, left ventricular ejection fraction; eGFR, estimated glomerular filtration rate; ABG, admission blood glucose; HBA_1_C, glycated hemoglobin; LDL-C, low-density lipoprotein cholesterol; HDL-C, high-density lipoprotein cholesterol; ACEI/ARB, angiotensin-converting enzyme inhibitor/angiotensin receptor blocker; SHR, stress hyperglycemia ratio

**Table 2:** Procedural characteristics of patients enrolled

	Total	Low SHR group (SHR < 1.191)	High SHR group (SHR ≥ 1.191)	*P* value
*Angiography*				
Number of diseased vessels				0.615
1-vessel disease	61 (48.0%)	28 (44.4%)	33 (51.6%)	
2-vessel disease	38 (29.9%)	19 (30.2%)	19 (29.7%)	
3-vessel disease	28 (22.0%)	16 (25.4%)	12 (18.8%)	
TIMI flow (Pre-PCI)				0.687
Grade 0	99 (78.0%)	51 (81.0%)	48 (75.0%)	
Grade 1	24 (18.9%)	10 (15.9%)	14 (21.9%)	
Grade 2	4 (3.1%)	2 (3.2%)	2 (3.2%)	
Grade 3	0	–	–	
Gensini score	58.00 (40.00, 80.00)	48.00 (38.00,80.00)	80.00 (49.00,84.00)	0.001
Syntax score	17.75 ± 6.03	16.35 ± 6.23	19.13 ± 5.53	0.009
*Intervention therapy*				
Total ischemic time (min)	422.53 ± 143.43	440.79 ± 122.29	404.55 ± 160.52	0.155
Stent implantation	127 (100.0%)	63 (100.0%)	64 (100.0%)	1.000
Number of stents	1.29 ± 0.54	1.30 ± 0.56	1.28 ± 0.52	0.832
Length of stents (mm)	34.28 ± 17.29	33.95 ± 18.38	34.59 ± 16.29	0.835
Diameter of stent (mm)	3.05 ± 0.41	3.04 ± 0.44	3.06 ± 0.37	0.796
Thrombus aspiration (%)	50 (39.4%)	18 (28.6%)	32 (50.0%)	0.018
GP IIb/IIIa inhibitor (%)	70 (55.1%)	33 (52.4%)	37 (57.8%)	0.594
TIMI flow ≥ 2 (Post-PCI)	120 (94.5%)	61 (96.8%)	59 (92.2%)	0.440

TIMI, thrombolysis in myocardial infarction; PCI, percutaneous coronary intervention; Total ischemic time, the period from symptom onset to reopening of infarction-associated artery; GP IIb/IIIa, glycoprotein IIb/IIIa; SHR, stress hyperglycemia ratio

As demonstrated in Tables 1 and 2, ABG, SHR, high-density lipoprotein cholesterol (HDL-C), and prevalence of thrombus aspiration were all higher in high SHR group compared to low SHR group patients. Additionally, as a continuous variable, SHR was also associated with ABG (r = 0.588, *p* < 0.001) and HDL-C (r = 0.220, *p* = 0.013). However, other parameters such as HbA1_C_, triglycerides, low-density lipoprotein cholesterol (LDL-C), and uric acid were all well matched between high SHR group and low SHR group.

### Association of SHR with myocardial injury and the severity of coronary artery disease

**Fig. 1:**
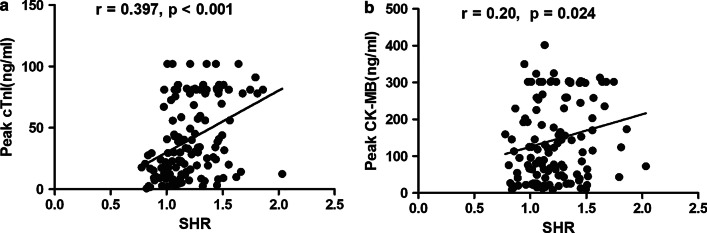
Association between SHR and peak cTnI (**a**) and peak CK-MB (**b**). SHR, stress hyperglycaemia ratio; cTnI, cardiac troponin I; CK-MB, creatine kinase muscle/brain subtype

As shown in Table 1, the low SHR group had significantly lower peak cTnI (20.62 (8.84, 40.64) vs 56.07 (25.43, 81.00), *p*  < 0.001) and peak CK-MB (89.38 (26.60, 202.60) vs 154.60 (54.18, 299.00), *p*  = 0.047) than the high SHR group. Additionally, as indicated in Fig. 1a, b, SHR had a positive correlation with peak cTnI (r = 0.390, *p* < 0.001) and peak CK-MB (r = 0.20, *p*  = 0.024).

**Fig. 2:**
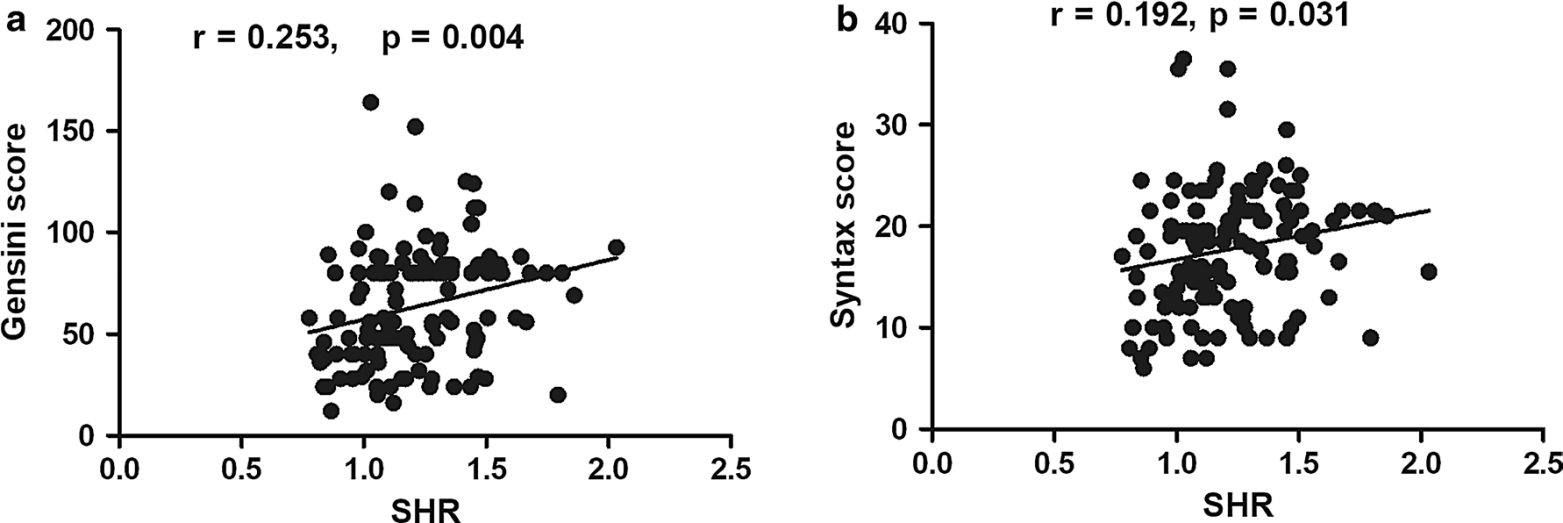
Associations of SHR with Gensini score (**a**) and Syntax score (**b**). SHR, stress hyperglycaemia ratio

Regarding the association of SHR with the severity of coronary artery disease, the low SHR group had lower Gensini score (48.00 (38.00, 80.00) vs 80.00 (49.00, 84.00), *p*  = 0.001) and Syntax score (16.35 ± 6.23 vs 19.13 ± 5.53, *p*  = 0.009) than the high SHR group. As a continuous variable, SHR also correlated positively with Gensini score (r = 0.253, *p*  = 0.004, Fig. 2a) and syntax score (r = 0.192, *p*  = 0.031, Fig. 2b).

### High SHR inhibited the early improvement of myocardial function

**Fig. 3:**
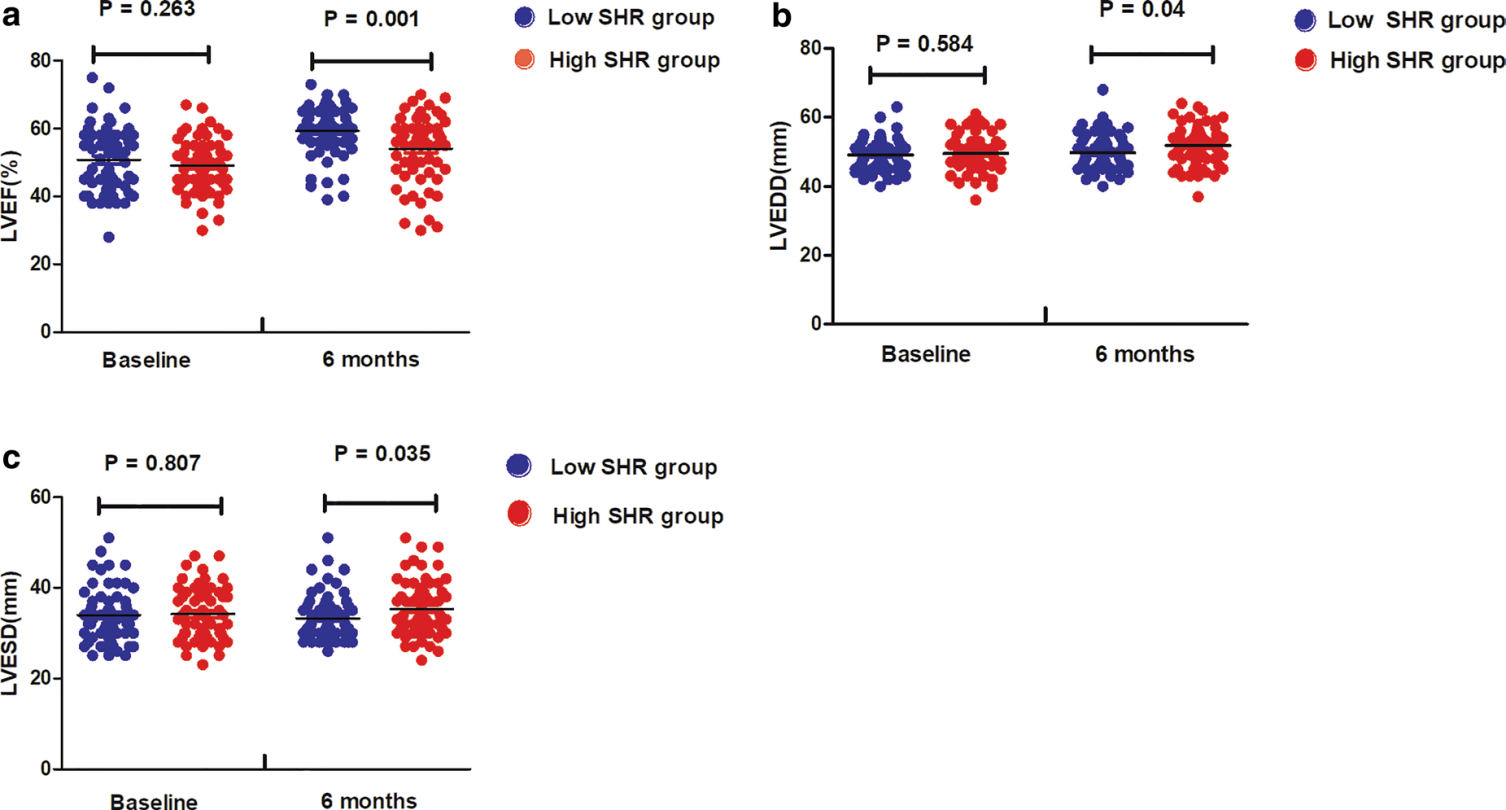
LVEF (**a**), LVEDD (**b**) and LVESD (**c**) in the low and high SHR groups at baseline and 6 months after infarction. LVEF, left ventricular ejection fraction; LVEDD, left ventricular end-diastolic diameter; LVESD, left ventricular end-systolic diameter

As shown in Fig. 3a, LVEF at 6 months after infarction in the low SHR group was significantly higher than that in the high SHR group (59.37 ± 7.33 vs 54.03 ± 9.64, *p*  = 0.001), even though there were no significant differences in baseline LVEF between the two subgroups (50.84 ± 9.41 vs 49.13 ± 7.67, *p*  = 0.263). Additionally, as shown in Fig. 3b, c, both LVEDD (49.84 ± 5.10 vs 51.81 ± 5.60, *p*  = 0.040) and LVESD (33.27 ± 5.03 vs 35.38 ± 6.05, *p*  = 0.035) at 6 months post-STEMI in the low SHR group were significantly lower than those in the high SHR group, with no difference in baseline LVEDD (49.0 8 ± 4.48 vs 49.55 ± 5.08, *p*  = 0.584) and LVESD (33.95 ± 5.85 vs 34.20 ± 5.68, *p*  = 0.807) between the two subgroups.

### Correlation between SHR and change in left ventricular geometric and functional properties

After calculating the change in echocardiography parameters, we examined the association between SHR and changes in echocardiography parameters. As presented in Tables 3, 4, and 5, SHR correlated well with δLVEF (beta = -8.753, 95% CI -15.040 to -2.466, *p*  = 0.007), δLVEDD (beta = 4.583, 95% CI 1.310 to 7.856, *p*  = 0.006), and δLVESD (beta = 5.497, 95% CI 1.303 to 9.690, *p*  = 0.011) in univariable linear regression analysis. After adjusting for other confounding variables (Age, sex, T2DM, total ischaemic time, number of diseased vessels, and variables with *p*  < 0.05 in univariable linear analysis) through multivariable linear regression analysis, SHR remained associated with δLVEF (beta = -9.825, 95% CI -15.168 to -4.481, *p*  < 0.0001), δLVEDD (beta = 4.879, 95% CI 1.725 to 8.738, *p*  = 0.003), and δLVESD (beta = 5.079, 95% CI 1.421 to 9.316, *p*  = 0.007), as indicated in Tables 3, 4, and 5, respectively.

**Table 3:** Correlation between change in LVEF and other variables using univariable and multivariable linear analysis

	Univariable linear analysis			Multivariable linear analysis		
	Beta	95%CI	*P* value	Beta	95%CI	*P* value
Age	− 0.052	− 0.196 to 0.092	0.473	0.063	− 0.060 to 0.186	0.310
Sex (Female)	− 6.627	− 11.182 to − 2.072	0.005	− 6.881	− 10.855 to − 2.907	0.001
T2DM	0.303	− 2.964 to 3.570	0.855	1.298	− 1.412to 4.008	0.345
BMI	0.658	0.054 to 1.262	0.033	0.449	− 0.047 to 0.944	0.076
SHR	− 8.753	− 15.040 to − 2.466	0.007	− 9.825	− 15.168 to − 4.481	< 0.0001
Total ischemic time	− 0.010	− 0.021 to 0.001	0.082	− 0.009	− 0.018 to < 0.0001	0.050
Number of diseased vessels	− 0.181	− 2.194 to 1.831	0.859	0.118	− 1.534 to 1.770	0.888
Baseline LVEF	− 0.518	− 0.681 to − 0.355	< 0.0001	− 0.568	− 0.714 to − 0.421	< 0.0001
Baseline LVEDD	− 0.043	− 0.379 to 0.294	0.802			
Baseline LVESD	0.231	− 0.046 to 0.508	0.102			

LVEF, left ventricular ejection fraction; Change in LVEF, change in LVEF was defined as (LVEF at 6 months after infarction) minus (baseline LVEF); T2DM, type 2 diabetes mellitus; BMI, body mass index; SHR, stress hyperglycemia ratio; LVEDD, left ventricular end-diastolic diameter; LVESD, left ventricular end-systolic diameter; CI, confidence interval

**Table 4:** Correlation between change in LVEDD and other variables using univariable and multivariable linear analysis

	Univariable linear analysis			Multivariable linear analysis		
	Beta	95%CI	*P* value	Beta	95%CI	*P* value
Age	− 0.005	− 0.08 to 0.071	0.905	0.016	− 0.059 to 0.091	0.671
Sex (Female)	0.487	− 1.961 to 2.935	0.695	− 0.909	− 3.295 to 1.477	0.452
T2DM	0.475	− 1.224 to 2.174	0.581	− 0.105	− 1.712 to 1.502	0.897
WBC	0.483	0.156 to 0.811	0.004	0.484	0.172 to 0.796	0.003
SHR	4.583	1.310 to 7.856	0.006	4.897	1.725 to 8.069	0.003
Total ischemic time	0.002	− 0.004 to 0.008	0.444	0.004	− 0.002 to 0.009	0.170
Number of diseased vessels	− 0.607	− 1.650 to 0.435	0.251	− 0.312	− 1.290 to 0.667	0.529
Baseline LVEF	− 0.090	− 0.186 to 0.006	0.066			
Baseline LVEDD	− 0.345	− 0.510 to − 0.181	< 0.0001	− 0.380	− 0.538 to − 0.221	< 0.0001

LVEDD, left ventricular end− diastolic diameter; Change in LVEDD, change in LVEDD was defined as (LVEDD at 6 months after infarction) minus (baseline LVEDD);T2DM, type 2 diabetes mellitus; WBC, white blood cell; SHR, stress hyperglycemia ratio; LVEF, left ventricular ejection fraction; CI, confidence interval

**Table 5:** Correlation between change in LVESD and other variables using univariable and multivariable linear analysis

	Univariable linear analysis			Multivariable linear analysis		
	Beta	95%CI	*P* value	Beta	95%CI	*P* value
Age	− 0.017	− 0.113 to 0.079	0.722	− 0.007	− 0.092 to 0.078	0.872
Sex (Female)	1.339	− 1.779 to 4.457	0.397	− 0.255	− 2.964 to 2.455	0.853
T2DM	− 0.585	− 2.755 to 1.585	0.595	− 0.988	− 2.859 to 0.884	0.298
HDL− C	4.661	0.946 to 8.376	0.014	2.633	− 0.643 to 5.909	0.114
WBC	0.442	0.017 to 0.868	0.042	0.390	0.036 to 0.743	0.031
SHR	5.497	1.303 to 9.690	0.011	5.079	1.421 to 8.738	0.007
Total ischemic time	0.007	0.001 to 0.015	0.051	0.007	0.001 to 0.013	0.024
Number of diseased vessels	− 0.941	− 2.269 to 0.387	0.163	− 0.163	− 1.286 to 0.959	0.774
Baseline LVEF	− 0.016	− 0.141 to 0.108	0.795			
Baseline LVESD	− 0.570	− 0.726 to − 0.414	< 0.0001	− 0.566	− 0.713 to − 0.419	< 0.0001

LVESD, left ventricular end-systolic diameter; Changer in LVESD, change in LVESD was defined as (LVESD at 6 months after infarction) minus (baseline LVESD);T2DM, type 2 diabetes mellitus; HDL, high density lipoprotein cholesterol; WBC, white blood cell; SHR, stress hyperglycemia ratio; LVEF, left ventricular ejection fraction; CI, confidence interval

## Discussion

In the present research, we demonstrated that the novel glycaemic index SHR correlated positively with myocardial injury as well as the severity of coronary artery disease. Subsequently, we confirmed for the first time that SHR is a major and independent predictor for left ventricular negative remodelling after first-time, acute, anterior STEMI.

Stress hyperglycaemia, as a useful biomarker, correlates well with adverse outcomes following AMI [10, 12]. However, there has been great confusion in the detection of stress hyperglycaemia and concerns regarding the methodological difficulties in measuring the degree of stress hyperglycaemia [15, 16]. Most previous studies mainly depended on ABG to measure the degree of stress hyperglycaemia without considering patients’ previous glycaemic statuses [10]. Additionally, a great deal of evidence has demonstrated that relative hyperglycaemia, the magnitude of an acute glycaemic increase from the baseline level, could be superior to the ABG level when assessing stress hyperglycaemia [10, 11, 15, 16]. SHR, a novel index of relative hyperglycaemia, was proposed by Roberts et al. and is defined as ABG/eAG [11]. Our present research and previous studies all indicated that patients in the high SHR group may have higher ABG levels and are more likely to undergo thrombus aspiration [12, 17]. SHR had no correlation with HbA1c in our present research, which was consistent with a previous study by Giancarlo Marenzi et al. [17].

Recently, a great deal of evidence has suggested that stress hyperglycaemia correlates positively with the degree of myocardial injury. For example, Timmer et al. and Jumaily et al. reported that ABG, the indicator of stress hyperglycaemia, correlated positively with cardiac damage measured by creatinine kinase/troponin level [18, 19]. In addition, Eitel et al. further confirmed that there was a stepwise relationship between glycaemic status on admission and infarct size quantified by cardiovascular magnetic resonance imaging (CMRI) [20]. For SHR and myocardial injury, mounting evidence has revealed that there was a graded relationship between SHR and peak cTnI [12, 17]. Extending previous studies, our present research suggested that peak cTnI and peak CK-MB were both significantly lower in the low SHR group than in the high SHR group. We also confirmed that SHR had a positive association with peak cTnI and peak CK-MB. Regarding stress hyperglycaemia and the severity of coronary artery disease, Qin et al. reported that the Gensini score of patients in the stress hyperglycaemia group was significantly higher than that of patients in the group without stress hyperglycaemia [21]. For SHR, our present research and a previous study all confirmed that SHR correlated well with the severity of coronary artery disease quantified by the Gensini score and Syntax score [12].

Subsequently, we investigated the association between stress hyperglycaemia and left ventricular negative remodelling after STEMI. Ishihara et al. reported that the ABG level correlated well with impaired pre-discharge LVEF in AMI patients [6]. Additionally, Bauters et al. confirmed that stress hyperglycaemia (defined as ABG > 7 mmol/L) remained associated with changes in left ventricular end-diastolic volume after multivariable analysis, which suggested that stress hyperglycaemia was an independent predictor of cardiac remodelling post-infarction [5]. In addition to clinical trials, Xie et al. demonstrated that acute hyperglycaemia could suppressed left ventricular diastolic function by inhibiting peroxisome proliferator-activated receptor-γ coactivator (PGC-1α) and autophagic flux in a mouse model [22]. Extending prior research, our present research reported that SHR, as a novel measurement of stress hyperglycaemia, correlated well with δLVEF, δLVEDD, and δLVESD after multivariate linear regression analysis, which further suggested SHR has a significant association with left ventricular negative remodelling.

Although, the potential mechanisms underlying the association between SHR and left ventricular negative remodelling are not fully understood in patients following AMI. There are several possible explanations according to previous studies. First, stress hyperglycaemia could increase the secretion of inflammatory and vasoconstrictive factors [15, 22]. Second, acute hyperglycaemia could abolished cardio-protection exerted by remote ischaemic preconditioning [23]. Third, stress hyperglycaemia could suppress left ventricular diastolic function by downregulating PGC-1α and inhibiting autophagic flux, which is reported by Xie et al. [22].

Although our present study demonstrated that SHR was associated with adverse left ventricular remodelling for the first time, some limitations should be acknowledged. First, this is a study at a single centre with a relatively small sample size. Hence, our findings need to be further verified by future multi-centre studies with large sample size. Second, we only recruited first-time, acute, anterior STEMI patients treated by primary PCI successfully within 12 h. Therefore, our findings may be unsuitable for general acute coronary syndrome patients. Third, Our present study mainly assessed the relationship between SHR and left ventricular negative remodeling after STEMI, without investigating normal range/distribution and therapeutic utility of SHR. Therefore, future studies are needed to investigate these parts.

## Conclusions

Our present research demonstrated that SHR, a novel index of relative hyperglycaemia, provides a major and independent predictive value for adverse left ventricular remodelling, even when the baseline left ventricular measurements were taken into account. Therefore, we may estimate the risk of left ventricular negative remodelling in STEMI patients by calculating SHR in the future, which is a widely available, non-invasive, and relatively inexpensive methods. However, before putting it into routine clinical practice, future studies with large sample size are still required.

## Data Availability

The data used to support the findings of this study are included within the article.

## Abbreviations

PCI: Percutaneous coronary intervention
STEMI: ST-segment elevation myocardial infarction
ABG: Admission blood glucose
SHR: Stress hyperglycaemia ratio
eAG: Estimated average glucose
HbA1c: Glycated haemoglobin
AMI: Acute myocardial infarction
LVEF: Left ventricular ejection fraction
AA: Aplastic anemia
MDS: Myelodysplastic syndromes
ACEIs: Angiotensin-converting enzyme inhibitors
ARBs: Angiotensin receptor blockers
cTnI: Cardiac troponin I
CK-MB: Creatine kinase muscle/brain subtype
BMI: Body mass index
TIMI: Thrombolysis in myocardial infarction
LVESD: Left ventricular end-systolic diameter
LVEDD: Left ventricular end-diastolic diameter
T2DM: Type 2 diabetes mellitus
HDL-C: High-density lipoprotein cholesterol
LDL-C: Low-density lipoprotein cholesterol
CMRI: Cardiovascular magnetic resonance imaging
PGC-1α: Peroxisome proliferator-activated receptor-γ coactivator
